# Intraoperative anti-inflammatory drugs combined with no drainage after MIS-TLIF in the treatment of recurrent lumbar disc herniation: an RCT

**DOI:** 10.1186/s13018-020-02155-x

**Published:** 2021-01-07

**Authors:** Jinpeng Du, Junsong Yang, Liang Yan, Lequn Shan, Wentao Wang, Yong Fan, Dingjun Hao, Dageng Huang

**Affiliations:** Department of Spine Surgery, Honghui Hospital, Xi’an Jiaotong University, Friendship East Road, Xi’an, 710054 Shaanxi Province China

**Keywords:** MIS-TLIF, Lumbar disc herniation, Nerve root pain, Gelatin sponge

## Abstract

**Background:**

Minimally invasive-transforaminal lumbar interbody fusions (MIS-TLIF), in which the nerve root pain is caused by early postoperative edema reaction, is a common clinical complication. However, there is no effective method to solve this problem. We aimed to use gelatin sponge impregnated with mixed anti-inflammatory drugs combined with no drainage after MIS-TLIF to optimize postoperative effect in the treatment of recurrent lumbar disc herniation (LDH).

**Methods:**

From June 2018, the middle-aged patients (45–60 years old) with recurrent LDH were recruited. Included patients were treated with MIS-TLIF surgery, and no drainage tube was placed after surgery. All patients were randomly divided into intervention group (gelatin sponge impregnated with mixed anti-inflammatory drugs) and control group (saline was immersed in gelatin sponge as a control).

**Results:**

The intervention group included 63 cases, and the control group included 65 cases. The length of hospital stays and bedridden period in the intervention group were significantly lower than those in the control group (*P* < 0.05). The VAS score of low back pain in the intervention group was significantly lower than that of the control group at postoperative days 1–6 (*P* < 0.05, for all). The VAS scores of leg pain in the intervention group at postoperative days 1–9 were statistically lower than the control group (*P* < 0.05, for all).

**Conclusions:**

Application of gelatin sponge impregnated with mixed anti-inflammatory drugs combined with no drainage after MIS-TLIF can significantly further optimize the surgical effect of recurrent LDH and shorten the bedridden period and hospital stays, to achieve the purpose of early rehabilitation.

**Trial registration:**

China Clinical Trial Registration Center, ChiCTR1800016236. Registered on May 21, 2018, http://www.chictr.org.cn/listbycreater.aspx

## Introduction

Minimally invasive-transforaminal lumbar interbody fusions (MIS-TLIF), in which the nerve root pain is caused by early postoperative edema reaction, is a common clinical complication, and according to previous literature, about 21% of patients with lumbar disc herniation have different degrees of postoperative low back pain and leg pain, and the average VAS score on the first day after surgery was 3.89 ± 1.75 [1]. The reason is that although the surgical intervention relieves the mechanical compression, the operation will inevitably aggravate the inflammatory chemical stimulus around the local nerve root [2, 3]. Moreover, the operating space under the channel is small, and the mechanical pull of the nerve hook and other operations are easy to damage the nerve root [4]. After the fibrous ring was cut during the operation, more contact surfaces of the nucleus pulposus center were exposed to the bloodstream immune environment, resulting in an autoimmune immune response, which together with local inflammatory chemical stimulation affected the pain relief after surgery [5]. Commonly used treatments are postoperative dehydration, administration of nonsteroidal anti-inflammatory drugs, and intravenous glucocorticoids. Even strong analgesic drugs are added in some patients, but the effect is limited [6].

At present, there is no effective method to solve nerve root pain caused by early postoperative edema reaction after MIS-TLIF. We thought of using gelatin sponge impregnated with mixed anti-inflammatory drugs to focus on it. The mixed drug used in this study was a mixture of dexamethasone, and vitamin B12 injection. Considering that the concentration of these drugs is difficult to maintain, the gelatin sponge is used to impregnate with these drugs without drainage after the operation. Try to slowly release the mixed drugs around the nerve root to extend the anti-inflammatory time.

Recent studies have shown that after MIS-TLIF, the drainage tube have not to be routinely left without any adverse events, and it will not affect the postoperative recovery of the patient due to the pain caused by the drainage tube around the wound [7, 8]. Therefore, the concentration of mixed drugs and the duration of drug action will not be reduced by drainage, which can reduce the risk of bias and improve the accuracy of research.

## Materials and methods

### Study design and participants

#### Inclusion and exclusion criteria

1. Inclusion criteria: [1] recurrent LDH [2]; conservative treatment is ineffective, with surgical indications [3]; middle-aged patients (age 45–60 years) [4]; lateral and central LDH without calcification, suitable for MIS-TLIF surgery [5]; single-segment LDH, and symptoms of one lower extremity [6]; reexamination of CT to exclude screw-related neurological complications; and [7] without other diseases that may cause leg pain
2. Exclusion criteria: [1] complicated with intervertebral space infection or postoperative wound infection [2]; those with huge disc herniation, difficult to remove under the channel, stenosis of the central tube, and extreme LDH [3]; patients with low pain threshold or psychological instability that cannot cooperate with evaluation [4]; allergy to intraoperative mixed drugs [5]; postoperative usage of analgesics or patient-controlled analgesia pump [6]; pregnant patients [7]; patients with incomplete clinical data or lost follow-up; and [8] patient rejected to sign the informed consent

### Recruitment and grouping

Starting from June 2018, middle-aged patients (45–60 years old) with recurrent LDH and surgical indications were recruited from this research center according to the pre-registered clinical research plan. Finally, 128 patients were included in the study and received MIS-TLIF surgery after screening, and no drainage tube is placed after surgery. All included patients were randomly divided into the intervention group (gelatin sponge impregnated with mixed anti-inflammatory drugs) and control group (saline was immersed in gelatin sponge as a control). Randomization was performed using sealed envelopes, a 20-number-per block randomization. The envelopes were consecutively numbered. The outcome of distribution remained unknown to both the patient and the surgeon until the patient’s written consent was obtained. An assistant blinded to the grouping independently collected baseline characteristics and clinical data of patients. The protocol of study has been approved by the Ethics Committee of Institution (approval number: 2018068) and successfully registered at China Clinical Trial Registration Center (ChiCTR1800016236).

### Surgical procedure

#### Intervention group

**Fig. 1:**
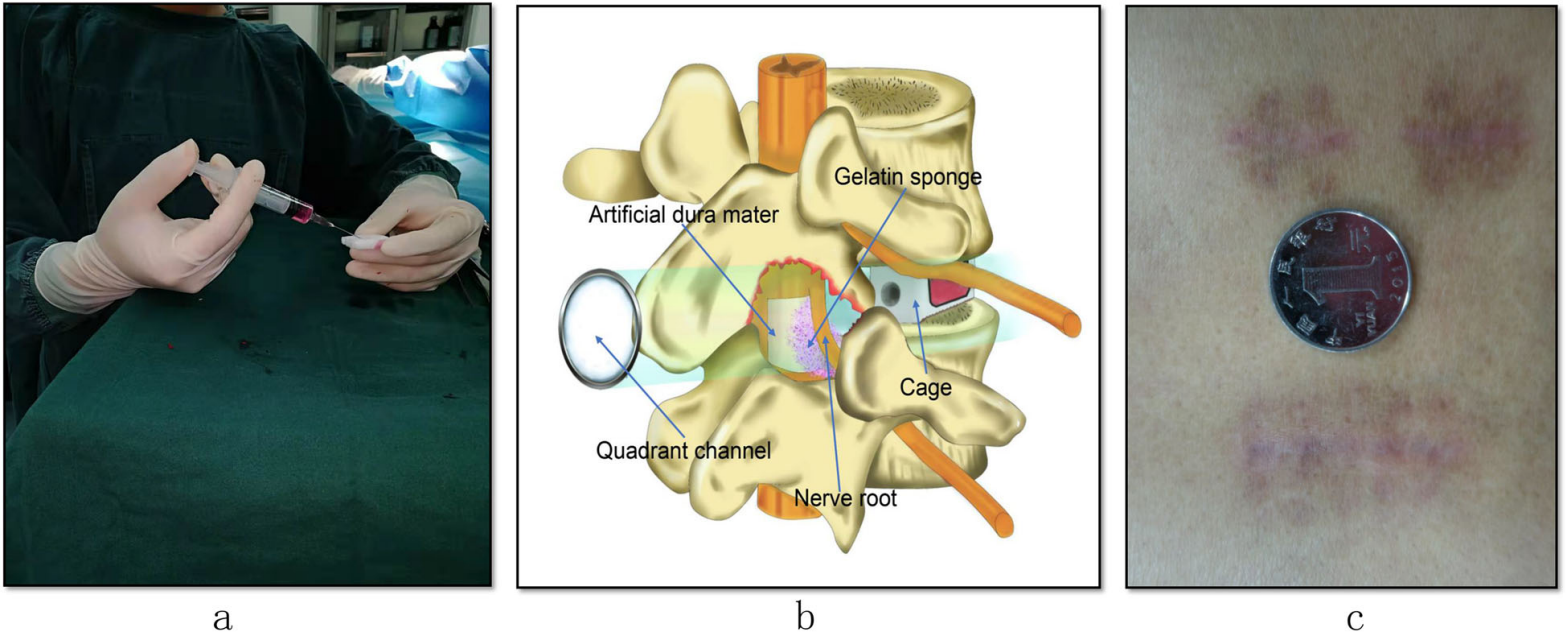
**a** The mixture of 2 drugs was injected into the gelatin sponge using a syringe. **b** The location of the intraoperative gelatin sponge and artificial dura mater. **c** The picture of healing wound

The intervention group is the conventional percutaneous pedicle insertion and decompression under the microendoscopy. Before closing the wound, place half of a piece of gelatin sponge (3 cm × 2 cm × 0.5 cm) impregnated with a mixture of 2 drugs (approximately 2.5 mL) around the nerve root (Fig. 1). The proportion of the mixture was 3 mL dexamethasone injection (1 mL:5 mg) plus 2 mL vitamin B12 injection (2 mL:0.5 mg). The dura mater and the dorsal side of the nerve root cover the artificial dura mater. Find a small bleeding point in the muscle and carefully stop the bleeding with an electrocoagulation knife. No drainage tube is placed.

#### Control group

After decompression and fixation, place half of a piece of gelatin sponge (3 cm × 2 cm × 0.5 cm) impregnated with 2.5 ml of normal saline on the ventral side of the nerve root. Other procedures were the same as those in the intervention group, and no drainage tube was placed after the operation.

### Postoperative treatment and care

All patients had the same postoperative treatment and care plan. Both groups were not routinely given intravenous or oral dehydration drugs, glucocorticoids, and non-steroidal anti-inflammatory drugs.

### Assessment criteria

1. Primary outcome measurements: visual analog scale (VAS) scores for low back pain and leg pain preoperatively and on postoperative days 1–10, and postoperative bedridden period and postoperative hospital stays.
2. Secondary outcome measures: Japanese Orthopaedic Association (JOA) score preoperatively and on postoperative days 3 and 6; Results of the satisfaction questionnaire at the 72nd hour after surgery (divided into four items: very satisfied, satisfied, just so so, and not satisfied).

#### Statistical analysis

Relevant clinical data were processed and analyzed using statistical software package SPSS 20.0 (SPSS, Inc, Chicago, IL, USA). For comparison of enumeration data, χ^2^ test was used. For comparison of continuous variable, a normality test (Shapiro-Wilk test) was performed first. If the normal distribution was met, an independent sample *t* test should be used, and the mean value was expressed as the mean ± SD. The significance level was set to *α* = 0.05.

## Results

### Comparison of baseline characteristics

After screening, a total of 128 peoples were included in the study and divided into an intervention group (63 cases) and a control group (65 cases). No patients in the intervention group withdrew from the trial. All patients have collected complete clinical research data. Baseline characteristics as shown in Table 1, the symptom duration and number of herniation levels in the two groups of patients were not found to be statistically different after statistical analysis (*P* > 0.05). Statistical analysis of the surgical time and blood loss between the two groups did not find significant differences (*P* > 0.05).

**Table 1:** Baseline characteristics

Parameter	Intervention group	Control group	*P* value
Number of patients	63	65	
Male patients, number (%)	36(57.1)	34 (52.3)	0.771
Age, years	52.3 ± 4.3	53.0 ± 4.4	0.580
BMI, kg/m^2^	24.1 ± 2.0	24.4 ± 2.3	0.632
Symptom duration, months	4.9 ± 8.5	4.5 ± 9.1	0.876
Surgical time, minutes	184 ± 22	179 ± 22	0.435
Intraoperative blood loss	127 ± 35	121 ± 38	0.572
Number of herniation levels			
L3–L4, *n*	2	3	0.149
L4–L5, *n*	28	32	
L5–S1, *n*	33	30	

**Fig. 2:**
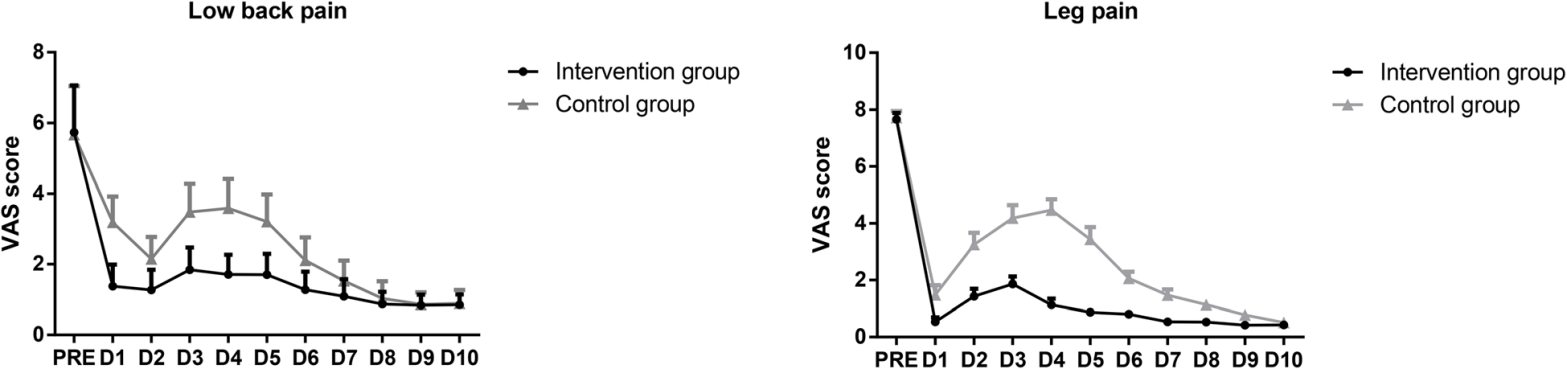
Short-term visual analog scale (VAS) score of leg and low back pain from preoperatively (PRE) to postoperative day (POD) 10

There was no significant difference in VAS scores of low back pain and leg pain between two groups (*P* > 0.05). The VAS scores of postoperative low back pain and leg pain in two groups were significantly lower than those before surgery, and the difference was statistically significant (*P* < 0.05). The VAS score of low back pain in the intervention group was significantly lower than that of the control group at postoperative days 1–6, and the difference was statistically significant (*P* < 0.05, for all). The VAS scores of leg pain in the intervention group were statistically lower than those in the control group at postoperative days 1–9 (*P* < 0.05, for all). It is suggested that the intervention group can significantly alleviate the pain of low back pain and leg pain after operation compared with the control group. See Table 2 for details, and summarize them into a line chart, as shown in Fig. 2.

**Table 2:** Visual analog scale scores for low back pain and leg pain from preoperatively to postoperative day 10

	Low back pain			Leg pain		
Parameter	Intervention group	Control group	*P* value	Intervention group	Control group	*P* value
PRE	5.74 ± 3.15	5.67 ± 3.27	0.940	7.69 ± 2.24	7.76 ± 2.31	0.916
POD1	1.38 ± 1.48^*^	3.19 ± 1.71^*^	**<0.0001**	0.51 ± 0.87^*^	1.57 ± 1.97^*^	**< 0.0001**
POD2	1.27 ± 1.36^*^	2.15 ± 1.49^*^	**0.038**	0.69 ± 1.06^*^	3.14 ± 2.35^*^	**< 0.0001**
POD3	1.84 ± 1.50^*^	3.48 ± 1.89^*^	**0.002**	1.68 ± 1.55^*^	4.00 ± 2.47^*^	**< 0.0001**
POD4	1.71 ± 1.33^*^	3.59 ± 1.94^*^	**<0.0001**	1.52 ± 1.40^*^	4.08 ± 2.19^*^	**< 0.0001**
POD5	1.70 ± 1.41^*^	3.21 ± 1.83^*^	**<0.0001**	0.71 ± 0.54^*^	3.71 ± 2.46^*^	**< 0.0001**
POD6	1.28 ± 1.21^*^	2.11 ± 1.55^*^	**0.044**	0.69 ± 0.47^*^	2.06 ± 1.38^*^	**< 0.0001**
POD7	1.09 ± 1.16^*^	1.53 ± 1.36^*^	0.234	0.43 ± 0.31^*^	1.33 ± 1.12^*^	**0.001**
POD8	0.87 ± 0.82^*^	1.03 ± 1.16^*^	0.584	0.38 ± 0.39^*^	0.77 ± 0.63^*^	**0.013**
POD9	0.84 ± 0.71^*^	0.86 ± 0.82^*^	0.928	0.41 ± 0.37^*^	0.75 ± 0.68^*^	**0.038**
POD10	0.85 ± 0.69^*^	0.89 ± 0.90^*^	0.864	0.39 ± 0.35^*^	0.53 ± 0.47^*^	0.248

*PRE* preoperative, *POD* postoperative day

*There is a significant difference compared with preoperatively

As shown in Table 3, the length of stay and bedridden period of the intervention group were significantly lower than those of the control group, and the differences were statistically significant (*P* < 0.0001). It is suggested that intervention measures can allow patients to walking exercises for rehabilitation early and reduce hospital stays.

**Table 3:** Postoperative length of stay and bedridden period

	Intervention group		Control group	*P* value
Postoperative length of stay, days	5.6 ± 0.8		7.2 ± 1.4	**< 0.0001**
Bedridden period, days	2.4 ± 0.5		3.1 ± 0.6	**< 0.0001**

There was no statistical difference in the JOA score between the two groups on pre-operation and postoperative day 3 (*P* > 0.05). The postoperative JOA scores of the two groups were significantly higher than those pre-operation, with a statistical difference (*P* < 0.05). And the JOA score of the intervention group on postoperative day 6 was higher than that of the control group, and the difference was statistically significant (*P* < 0.0001). It suggested that the intervention group was better than the control group in the improvement of patients’ postoperative function. See Table 4 for details.

**Table 4:** Measurement outcomes of Japanese Orthopaedic Association Score

Groups	Number	PRE	POD3	POD6
Intervention	63	10.7 ± 2.2	15.8 ± 2.6^*^	19.3 ± 2.8^*^
Control	65	10.6 ± 2.0	14.9 ± 3.3^*^	17.2 ± 3.4^*^
*P* value	–	0.788	0.089	**< 0.0001**

*PRE* preoperative *POD* postoperative day

*There is a significant difference compared with preoperatively

As shown in Table 5, for patient satisfaction at 72 h postoperatively, there was no statistical difference between two groups (*P* > 0.05). It is suggested that the minimally invasive operation gives patient sufficient satisfaction. Significantly effective rate in the intervention group was 46.0% and in the control group was 32.3%. However, comparison between groups of clinical effects on postoperative day 6 according to JOA score shows, there was no statistical difference between two groups (*P* = 0.112) (Table 6).

**Table 5:** Comparison of satisfaction in groups 72 h after operation

Groups	N	Very satisfied	Satisfied	Just so so	Not satisfied
Intervention	63	58	4	1	0
Control	65	48	13	3	1

**Table 6:** Comparison between groups of clinical effects on postoperative day 6 according to Japanese Orthopaedic Association Score

Groups	Number	Significantly effective	Effective	Invalid
Intervention	63	29	34	0
Control	65	21	44	0

*JOA score* Japanese orthopedic association score

There was one case (1.5%) in the control group occurred postoperative infection. Both groups of patients underwent postoperative computed tomography. There were no cases of breakage of pedicle screw and titanium rod or cage displacement.

## Discussion

MIS-TLIF technology has less blood loss during operation, and no drainage tube can be placed after operation. Xu et al. [8] study the necessity of indwelling tube after MIS-TLIF; in the drainage group, 28 patients had an average drainage volume of <50 ml in the first 24 h after surgery. The complications such as postoperative incision infection and hematoma compression did not increase in the non-drainage group, and their postoperative bedridden period and hospital stay were significantly reduced, postoperative low back pain significantly relieved. It is proved that not placing a drainage tube routinely does not have great risks, and its clinical benefits far exceed the conventional placement of drainage tubes.

The drug used in this study was a mixture of dexamethasone and vitamin B12. Dexamethasone can inhibit the excitability of nerve endings, improve local blood circulation, make local metabolites easy to be removed from the blood circulation, alleviate local acidosis, and help reduce inflammation. Dexamethasone can significantly expand the pharmacological effects of vitamin B12. Its molecular mechanism is that dexamethasone has a large molecular size and a complex spatial structure, which affects the release and absorption of vitamin B12 [9]. Vitamin B12 can provide nutrition for the nerve tissue, reduce abnormal discharge of damaged nerves, and relieve pain indirectly [10]. The combination of two drugs accords with pharmacokinetics and drug individualization principles; the safety, feasibility, and practical value were proved by our previous study [11].

However, concentration of the combination of these drugs is difficult to maintain during surgery. Absorptive gelatin sponge is a semi-synthetic material. Its unique sponge-like structure has protective effects on nerve roots [12], and its collagen properties have a good effect on preventing adhesions around the dura mater and nerve roots after decompression [13]. At the same time because of its good hemostatic effect is often used in spinal surgery. Previous researchers have impregnated gelatin sponges with hemostatic drugs and found that gelatin sponges have strong water absorption and slow release of tranexamic acid, which can significantly reduce postoperative wound bleeding in posterior lumbar spine surgery, and do not increase the risk of wound infection and epidural hematoma [14, 15]. Gelatin sponges are indeed a good carrier for impregnating drugs.

After research, we found that this method has a good effect on reducing postoperative root pain, and the effect of promoting early recovery. There was no significant difference in the results of the satisfaction survey. Considering that patients who were informed by the surgeon before surgery has a certain psychological expectation; moreover, the neurological symptoms of patients after sufficient decompression are significantly relieved than before surgery.

In this study, we found that VAS scores of back pain and leg pain in the two groups began to increase on the second day after surgery (Fig. 2), indicating that radicular nerve root edema pain began to appear at this time, while the VAS score of the control group reached the peak on the 3rd to 4th days after surgery. It may be considered that the early rehabilitation exercise stimulated the radicular nerve root edema to worsen. The appearance of the peak VAS score in the intervention group means that early rehabilitation exercises will indeed stimulate further edema of the nerve root to a certain extent, but its peak value is significantly lower than that in the control group, indicating that the anti-inflammatory effect of the mixed drug is obvious, and the duration more than 72 h.

In addition, this new method has another obvious advantage. Compared with conventional postoperative routine intravenous or oral administration of various hormones and non-steroidal anti-inflammatory drugs, this study illustrates a cheap and low-cost method, which can greatly reduce the economic burden of patients.

With regard to the use of this new technology, the following points need special attention. First, there is a risk of the local drugs penetration into the dura mater [16]. According to our previous clinical experience, it is recommended to use artificial dura to attach to the surface of dura and then place a gelatin sponge impregnated with mixed drugs. Artificial dura mater cannot only prevent nerve root adhesion, but also slow down the spread of mixed drugs to the subdural space.

Although there are obvious differences in the relief of pain between the intervention group and the control group, some patients still have recurrent pain after surgery. Elderly patients may experience more drug dependence relative to younger patients [17], and individual differences in pain sensitivity must also be considered. In addition, it is not clear how long the effectiveness of mixed drugs lasts, or whether the mixing ratio is able to achieve the best results; therefore, further research is still needed. All in all, there are inevitably all kinds of biases, but the data of this study is reliable in general, and there is no possibility of false-positive results caused by obvious biases.

## Conclusions

Application of gelatin sponge impregnated with mixed anti-inflammatory drugs combined with no drainage after MIS-TLIF can significantly reduce short-term pain of low back pain and leg pain, and further optimize the surgical effect of recurrent LDH and shorten the bedridden period and hospital stay, to achieve the purpose of early rehabilitation. It can also reduce the cost of hospitalization and reduce the financial burden on patients.

## Data Availability

The datasets used and/or analyzed during the current study are available from the corresponding author on reasonable request.

## Abbreviations

MIS-TLIF: Minimally invasive - transforaminal lumbar interbody fusions
LDH: Lumbar disc herniation
VAS: Visual analog scale
JOA: Japanese Orthopaedic Association Scores
PRE: Pre-operation
POD: Postoperative day
